# Computer-Simulated Virtual Image Datasets to Train Machine Learning Models for Non-Invasive Fish Detection in Recirculating Aquaculture

**DOI:** 10.3390/s24175816

**Published:** 2024-09-07

**Authors:** Sullivan R. Steele, Rakesh Ranjan, Kata Sharrer, Scott Tsukuda, Christopher Good

**Affiliations:** The Conservation Fund Freshwater Institute, Shepherdstown, WV 25443, USA

**Keywords:** precision aquaculture, RAS, underwater imaging, artificial intelligence, computer vision

## Abstract

Artificial Intelligence (AI) and Machine Learning (ML) can assist producers to better manage recirculating aquaculture systems (RASs). ML is a data-intensive process, and model performance primarily depends on the quality of training data. Relatively higher fish density and water turbidity in intensive RAS culture produce major challenges in acquiring high-quality underwater image data. Additionally, the manual image annotation involved in model training can be subjective, time-consuming, and labor-intensive. Therefore, the presented study aimed to simulate fish schooling behavior for RAS conditions and investigate the feasibility of using computer-simulated virtual images to train a robust fish detection model. Additionally, to expedite the model training and automate the virtual image annotation, a process flow was developed. The ‘virtual model’ performances were compared with models trained on real-world images and combinations of real and virtual images. The results of the study indicate that the virtual model trained solely with computer-simulated images could not perform satisfactorily (mAP = 62.8%, F1 score = 0.61) to detect fish in a real RAS environment; however, replacing a small number of the virtual images with real images in the training dataset significantly improved the model’s performance. The M6 mixed model trained with 630 virtual and 70 real images (virtual-to-real image ratio: 90:10) achieved mAP and F1 scores of 91.8% and 0.87, respectively. Furthermore, the training time cost for the M6 model was seven times shorter than that for the ‘real model’. Overall, the virtual simulation approach exhibited great promise in rapidly training a reliable fish detection model for RAS operations.

## 1. Introduction

Land-based intensive fish farming methods such as recirculating aquaculture systems (RASs) are emerging as a sustainable approach to produce premium quality fish near the consumers [1,2]. Appropriate RAS management strategies are critical to maintain an optimal rearing environment for cultured fish [3]. Currently, precision technologies such as sensor networks, computer vision, Artificial Intelligence (AI), and Internet of Things (IoT) are being adopted in the aquaculture industries to improve yield, profitability, and production sustainability [4,5,6]. The harsh operating conditions, high maintenance cost, and limited power and data connectivity, however, present major challenges in large-scale commercial adaptation and affect the economic viability of incorporating advanced technology into coastal sea cage farming [7]. Such technologies can be relatively easily adopted in RASs, as these systems enable farmers to rear fish in a controlled environment. Recent studies indicate several applications of Machine Learning (ML)-assisted image classification and object detection for non-invasive biomass estimation [8], fish health and welfare monitoring [9,10], disease prediction [11], feed optimization [12], behavior monitoring [13], water quality optimization [14], and mortality monitoring [15] in RASs.

Despite numerous research efforts toward the development of AI- and ML-aided solutions for efficient RAS management, the scalability of this approach is still questionable [16]. ML techniques are highly data-intensive, and the precision and accuracy of the models primarily depend on the quality of the input data [17,18]. A widely adopted technique used to increase the data size and incorporate variability in the training dataset is augmentation [19]. A series of transformations (e.g., rotation, flip, brightness, saturation, etc.) are applied to the images, and these images are then added to the original training dataset to enhance model performance; however, these techniques can only be effective when sufficient input data are available to perform augmentations. This may exclude scenarios where image acquisition is difficult, costly, or hazardous but are relevant to computer vision problems [20]. Alternatively, computer simulation techniques can be adapted to mimic real-world conditions and generate virtual videos and/or images for model training. The feasibility of using virtual images for ML model training has been explored over the past decade, with an emphasis on semantic segmentation and object detection [21]. Studies report the use of synthetic training data for traffic sign recognition [22,23], pedestrian detection [17], and vehicle detection [24]. Similar approaches have also been explored in agricultural and environmental applications such as crop yield estimation [25], habitat monitoring [20,26], semantic part localization [27], and underwater inspection [28], and they have produced promising results.

Acquiring underwater images to train ML models presents a unique challenge, as the image quality is adversely affected by high turbidity, high population density, moving targets (i.e., fish), and their proximity to the camera [29,30]. Recent research has explored techniques such as Principal Component Fusion of Foreground and Background, and Weighted Wavelet Visual Perception Fusion to enhance underwater image quality [31,32]. However, accurately restoring the true colors of underwater scenes remains challenging, particularly in instances of significant color degradation caused by water absorption and scattering. Furthermore, manual image annotation prior to model training is a time-consuming and labor-intensive process and may introduce subjectivity and inaccuracy into the model [33]. Additionally, the data acquisition time frame highly relies upon the occurrence of the conditions for which the model is being trained. For instance, if an ML model is being trained to detect infrequently occurring high-mortality events, creating conditions like this for model development is both challenging and undesirable [15].

Using a virtually generated dataset simulated for a RAS environment may present a solution to these problems. This approach can assist in generating various scenarios (e.g., high mortality, disease conditions, etc.) for RASs and theoretically provide unlimited data for model training. Imaging conditions (e.g., camera location and orientation, fish density, water quality, etc.) can also be adjusted with a click of the mouse. Additionally, the annotation process can be automated for rapid and robust model development. Under this hypothesis, the presented study was conducted to explore the feasibility of computer simulation to generate virtual images and utilize these images for training in-tank fish detection models. The specific objectives of the study were as follows:

1. To virtually simulate a RAS environment and optimize the fish schooling pattern to attain high-quality virtual image data suitable for training a robust in-tank fish detection model.
2. To analyze the performance of a virtual image-trained fish detection model and compare its performance with a model trained with real-world data.

## 2. Materials and Methods

### 2.1. Virtual Simulation

#### 2.1.1. Fish Schooling

RAS-appropriate fish schooling was simulated in Blender (Blender Foundation, Amsterdam, The Netherlands), an open-source simulation and rendering software, using a Boids particle system. A Boids particle system can be used to simulate flocks, herds, swarms, and schools of various kinds of animals. The particles within the Boids system can be programmed to follow basic rules and behavior to emulate certain movement patterns [34].

**Figure 1:**
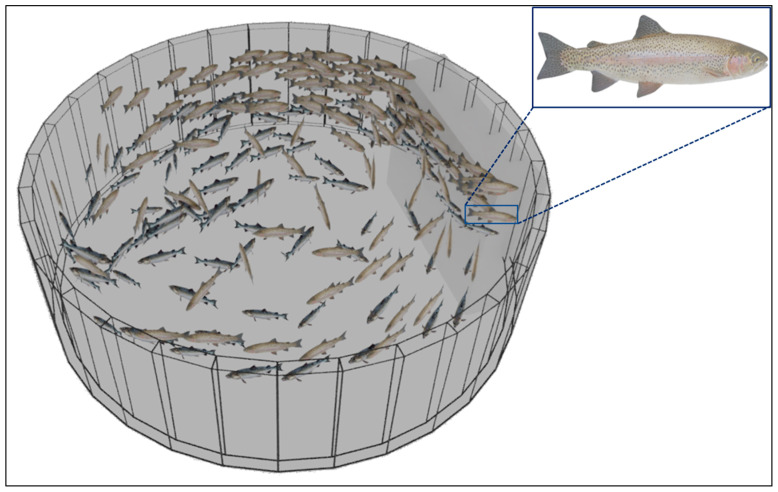
The Boids particle system used to mimic the schooling behavior of fish in a RAS tank. A cuboid attached to the tank wall acted as a virtual barrier to divert the Boids particles (i.e., fish, in inset) away from the camera.

The first step in the simulation process was to generate a virtual RAS tank. A hollow cylinder was created to mimic a fish tank so that fish schooling behavior and light conditions could be simulated within the tank. A 3D rainbow trout (*Oncorhynchus mykiss*) model (hereafter termed as ‘fish’, format: FBX; PoserWorld, Vanishing Point Inc., Orlando, FL, USA) with 5291 polygons was imported into the Blender environment. A unique fish was associated with each Boids particle to simulate the swimming pattern of fish in the virtual RAS tank (Figure 1). Collision avoidance was enabled so that collisions among the fish and between fish and tank walls/floor were avoided. Additionally, an invisible plane was used to cover the tank top to contain the fish within the virtual RAS tank. Two vortex forces were applied from the top and bottom of the tank, and their vortex intensities and orientations were adjusted to optimize fish movements and swimming patterns within the virtual RAS environment. The clockwise top vortex and counter-clockwise bottom vortex forces ensured the uniform vertical and horizontal distribution and circular motion of fish with reference to the tank axis. To solve the problem of fish swimming too close to the camera, a virtual ramp (represented by a cuboid in Figure 1) was introduced upstream to the camera near the tank wall. This barrier helped to divert the fish away from the camera, as illustrated in Figure 1.

#### 2.1.2. Underwater Environment

**Figure 2:**
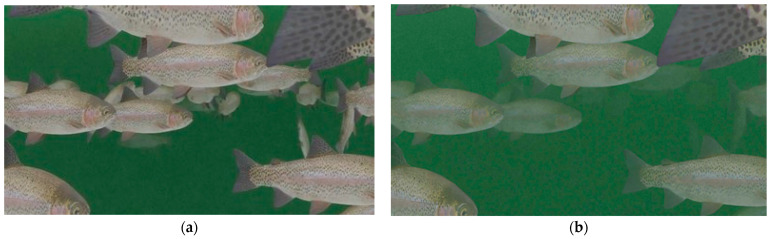
The training images generated for (**a**) low-turbidity and (**b**) high-turbidity conditions. The low-turbidity images had well-defined object features, whereas in turbid conditions, blurry object features can be observed.

The simulation aimed to mimic the swimming patterns of fish in RAS environment, realistically incorporating the effects of water quality and lighting on underwater visibility. Area light was adopted for the simulation to emulate the uniform lighting often seen in indoor RAS facilities. The Shading feature in Blender was applied to the virtual tank to simulate the turbid water quality seen in real-world RAS conditions. The Shader uses the material property and distance between the virtual camera and objects to evaluate the scattering or absorption of the light. As an object moves away from the camera, higher absorption and scattering of light create a darkening and blurring effect. The software allows users to adjust the distance associated with light scattering and absorption. This method was used to create the perception of turbidity (i.e., low or high) in the water to reflect real-world visibility conditions in the virtual RAS tank. The images generated for low-turbidity conditions had a higher number of fish in the foreground and well-defined object features compared to the images generated for high-turbidity conditions (Figure 2).

### 2.2. Validation Data Acquisition

The best-performing image dataset from our recent study [30] was utilized to analyze the performance of the virtual image-trained fish detection models in real-world scenarios. The images were collected in a 150 m^3^ fully recirculating, semi-commercial-scale growout tank located at The Conservation Fund’s Freshwater Institute (Shepherdstown, WV, USA). The tank was stocked with rainbow trout with a tank density at the time of data collection of 40 kg m^−3^. The image data were captured using a Raspberry Pi sensor (RPi, model: M23272M14, Arducam, Nanjing, China; focal Length: 2.72 mm, Aperture: F2.5, horizontal field of view (HFOV): 140°, and captured resolution: 1920 × 1080 px) in supplemental light conditions. The details of the data acquisition protocol are reported in [30].

### 2.3. Automated Image Annotation

**Figure 3:**
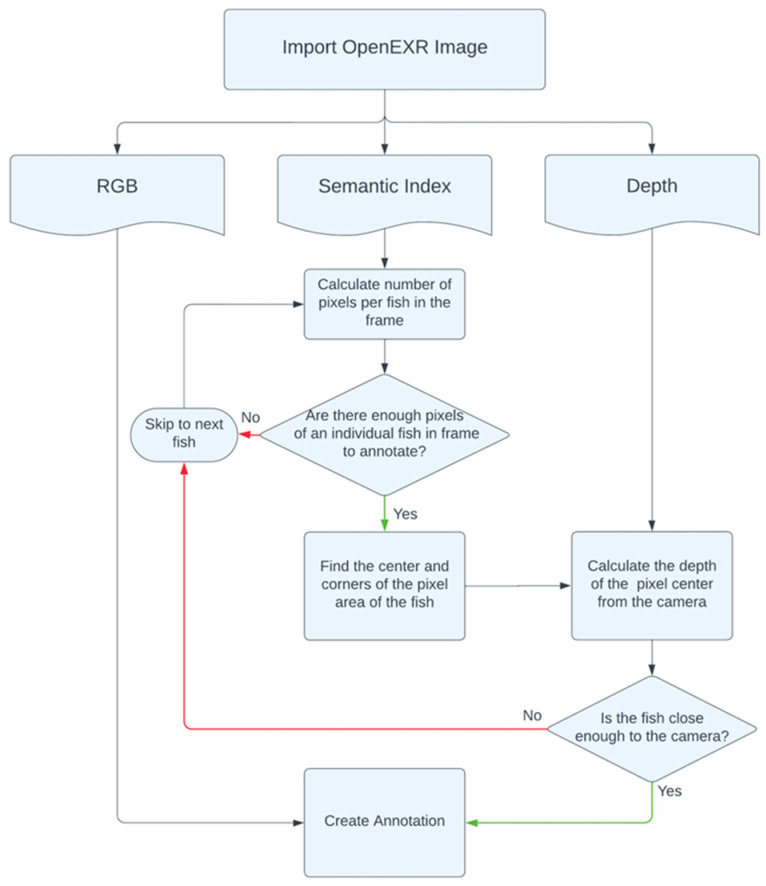
Algorithm process flow developed for automated annotation of simulated images.

**Figure 4:**
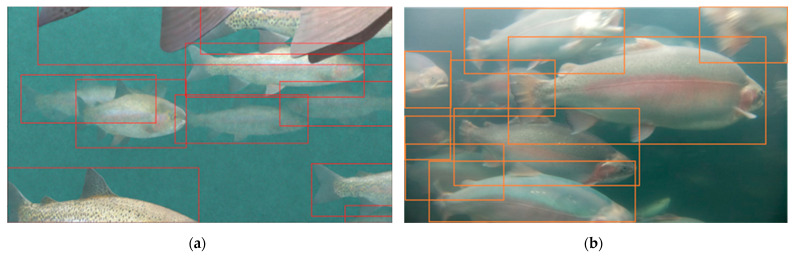
An automatically annotated (**a**) virtual image and (**b**) manually annotated real image, which were used to train the virtual model and real model, respectively. The rectangular boxes in the image represent annotated partial and whole fish in the image.

The virtual simulation model rendered layered images in OpenEXR format, which allowed us to store an arbitrary number of attributes in an image file. OpenEXR images can contain a combination of image channels, for instance, red (R), green (G), blue (B), luminance, chroma, depth, surface normal directions, and/or motion vectors. For this study, RGB, depth, and semantic index map layers were included in the output image (Figure 3). While RGB features assisted in visualizing the object of interest (i.e., fish) in the image, the semantic index map provided information on whether an individual pixel belonged to the object of interest (i.e., fish) or background (i.e., tank or water). If an object was identified as fish, the number of pixels for an individual fish was used to determine whether to include or exclude a fish for annotation. The selected fish was further checked for its distance from the camera, and pixel depth information retrieved from the depth map was used to exclude the farther-located blurry fish. Finally, based on the semantic information of the fish, the center of the volume, depth, and box coordinates of each fish was determined, and annotations were created. The time cost required to annotate the virtual training image was logged and compared with the manual annotation of real images (Figure 4).

The training dataset containing the image and corresponding annotation metadata was stored in Common Object in Context (COCO) format [35]. COCO formats the data by information, licenses, categories, images, and annotations in a JavaScript Object Notation (JSON) file. This format enables the storage of various annotation types, such as bounding boxes, segmentation masks, key points, and natural language descriptions. The COCO dataset was then imported into Roboflow (Roboflow, Inc., Des Moines, Iowa, USA) to train the fish detection model.

### 2.4. Model Training

A single-stage object detection model, YOLOv8 [36], was adopted for in-tank fish detection. Three different types of training datasets were used to train various fish detection models. The first model (hereafter termed the ‘virtual model’) was trained with simulation-generated virtual training images only, whereas the second model used real-world underwater imagery data acquired under RAS conditions to train the fish detection model (hereafter termed the ‘real model’), as described in Section 2.2. The third type of model (hereafter termed the ‘mixed model’) was trained with datasets containing both virtual and real images in varying proportions. Eight mixed models (M1, M2, M3, M4, M5, M6, M7, and M8) were trained with datasets containing both virtual and real images in proportions of 99:1, 98:2, 96:4, 94:6, 92:8, 90:10, 75:25, and 50:50, respectively (Table 1). Only real images were used for validation and test datasets so that the feasibility of using virtual image datasets to train fish detection models for real-world scenarios could be evaluated. Model training and model validation were performed using two computer vision tools (Roboflow, Inc., Des Moines, Iowa, USA; Ultralytics, Los Angeles, CA, USA). A proportion of 70:20:10 was maintained for the training, validation, and test dataset, and each model was trained up to 100 epochs.

### 2.5. Data Analysis

The model performances were analyzed in terms of mean average precision (mAP) at an intersection over union (IoU) threshold of 0.5 (i.e., mAP0.5) and F1 score as described by Flach and Kull (2015) [37]. Mean average precision (mAP0.5) was the mean of the precision under different recall values at an IoU of 0.5, whereas the F1 score was the harmonic mean of precision and recall. The real, mixed (M1–M8), and virtual models were first trained with different dataset sizes (100–1000 images at a step of 100 images), and the effect of data size on model performance was analyzed. Additionally, the preprocessed training images were augmented (2X) with brightness [±25%], exposure [±25%], saturation [±25%], and blur [±5%], and the performances of the augmented models (i.e., With Aug) were compared with that of the non-augmented model (i.e., No Aug). Finally, the performances and total training time costs of the optimized real, mixed, and virtual models were compared. The total time cost was evaluated as the sum of the annotation time (i.e., time required for image annotation) and model training time (i.e., time required to train a model).

## 3. Results and Discussion

### 3.1. Model Optimization

#### 3.1.1. Epoch and Data Size

**Figure 5:**
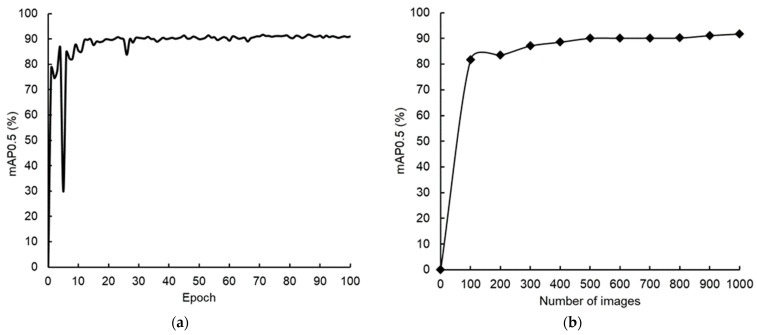
The mean average precision (mAP0.5) scores attained by the M6 mixed model (training dataset consisting of 90% virtual and 10% real images) trained with different (**a**) epochs and (**b**) data sizes.

While training the real and mixed models (M1–M8) for fish detection, a logistic increase in the mAP scores was observed up to 30 epochs. Further training the models up to 100 epochs slightly improved the mAP and reduced the losses; however, beyond 100 epochs, no improvement in model performance was observed (Figure 5a). Unlike real and mixed models, the virtual model training stopped after 60 epochs due to model overfitting and resulted in an increase in the loss values and degraded mAP scores for higher epochs. Similar mAP trends were observed while analyzing the performance of virtual, real, and mixed models trained with different data sizes. Increasing the data size beyond 1000 images failed to improve the model performance in terms of mAP. Additionally, training the model beyond the optimal data size and epochs may lead to model overfitting and result in higher computing and time costs [38]. Therefore, for further analysis, all models were trained with 700 images (70%), validated on 200 images (20%), and tested on 100 images (10%) up to 100 epochs.

#### 3.1.2. Fish Detection Model

**Figure 6:**
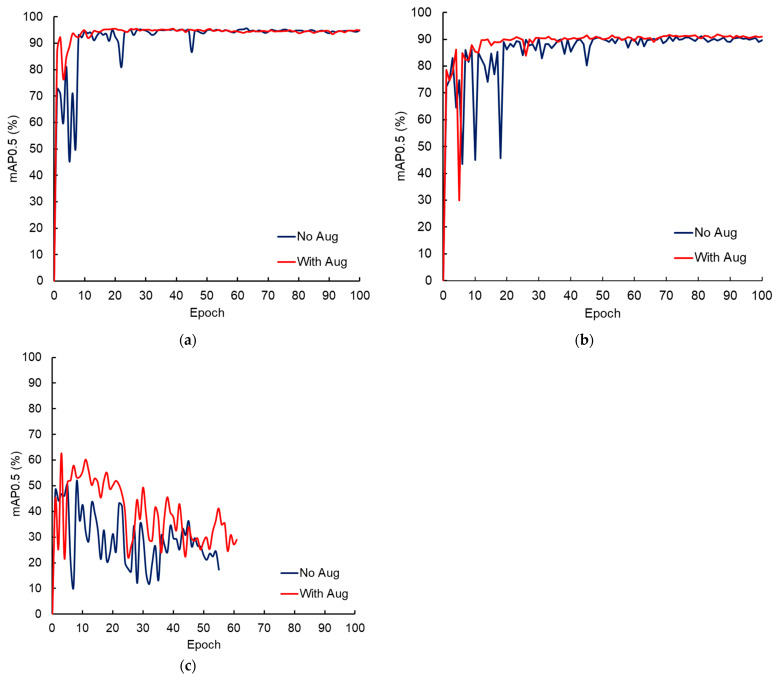
The effect of data augmentation on the mean average precision (mAP) score of (**a**) real, (**b**) mixed, and (**c**) virtual fish detection models.

While analyzing the impact of data augmentation on the performance of real and mixed models, we found that the augmentation apparently does not considerably affect the model performance. The model trained with an augmented dataset showed better performance during the initial stage of training; however, the effect of augmentation diminishes for a higher number of epochs (Figure 6a,b). Similar results were reported in our prior study conducted to study the effect of image data quality on fish detection model performance [30]. The difference in mAP score between models trained with augmented and non-augmented datasets was less than 1%. Unlike real and mixed models, data augmentation assisted in improving the performance of the virtual model (Figure 6c). The maximum mAP and F1 scores of the virtual model trained with a non-augmented dataset were 51.4% and 0.53, respectively. The augmented training dataset enhanced model performance and achieved mAP and F1 scores of 62.8%, and 0.61, respectively. Overall, data augmentation either improved or had no impact on model performance; however, augmentation did not negatively affect model performance. Therefore, to maintain consistency in the analysis, all datasets were augmented prior to the training, and pertinent results are presented in the following section.

### 3.2. Model Performance

**Figure 7:**
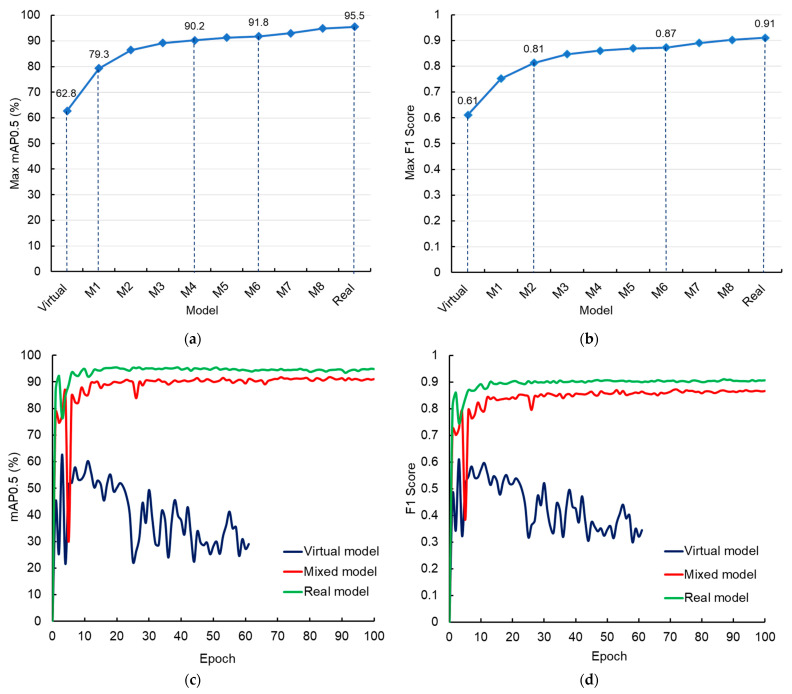
The maximum (**a**) mean average precision (mAP0.5) and (**b**) F1 scores attained by virtual, mixed (M1–M8), and real fish detection models and (**c**,**d**) performance comparison of M6 model trained with 90% virtual and 10% real images against the virtual and real models.

**Figure 8:**
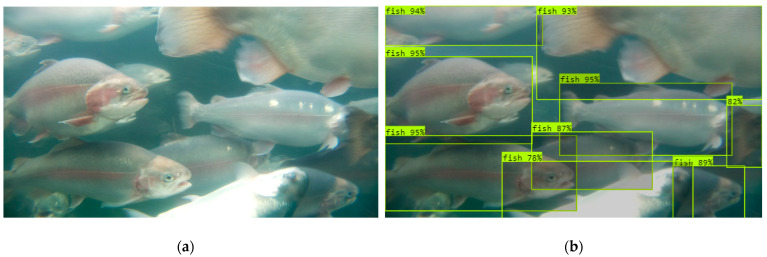

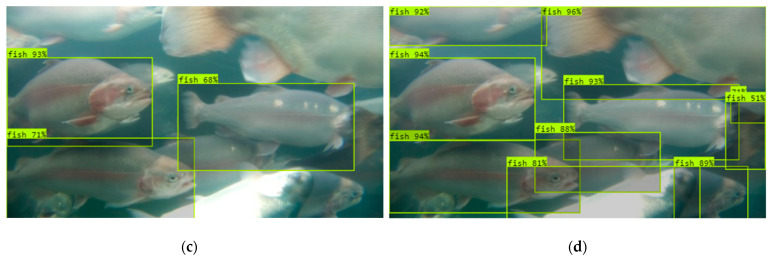
(**a**) A sample image acquired in a RAS environment and output images depicting the fish detected in the frame while deploying the (**b**) real model, (**c**) virtual model, and (**d**) mixed model (M6) to sample images.

The real model trained with underwater images acquired by the Rpi sensor performed satisfactorily in terms of in-tank fish detection. The maximum mAP value for the optimized real model was above 95% (Figure 7a). The real model also achieved high precision and recall, with an F1 score of 0.91 (Figure 7b). When deploying the model on an independent image dataset captured in a real RAS environment, the model accurately detected the whole fish. Additionally, the model also successfully detected partial fish with more than 50% area of their body visible in the frame (Figure 8b).

Unlike the real model, the virtual model trained solely with computer-simulated virtual images did not perform satisfactorily in real-world underwater environments. The maximum mAP and F1 scores for this model were 62.8%, and 0.61, respectively. The virtual model accurately detected the whole fish; however, it failed to detect the partial fish in the frame (Figure 8c). This resulted in an underprediction of the fish population by the virtual model.

While the model trained with virtual images alone could not perform satisfactorily, replacing small numbers of virtual images with real images significantly improved the model performance. Out of the 700 images used to train the virtual model, replacing 1% of the virtual images with real images resulted in a substantial improvement in the performance of the M1 mixed model. Replacement of seven virtual images with real images resulted in improvements in the mAP score, increasing from 62.8% to 79.3%, and F1 score, which increased from 0.61 to 0.75. The performance of the mixed model further improved as the proportion of real images in the training dataset was increased. The M4 mixed model trained with 658 virtual and 42 real images (virtual-to-real image ratio: 94:6) surpassed an mAP value of 90%. Replacing 10% of the virtual images with real images helped the M6 model to achieve a satisfactory mAP of 91.8% and an F1 score of 0.87. Additionally, the M6 model performed on par with the real model in terms of fish detection in real-world conditions and precisely detected the whole and partial fish in the frame (Figure 8d). Therefore, the M6 mixed model was adopted for further comparative analysis. A study conducted by Jelic et al. (2022) [39] to develop advanced driver-assistance system algorithms for autonomous vehicles reported similar findings and concluded that synthetic data can contribute to better detector performance until a certain ratio of real-world and synthetic data is reached.

### 3.3. Model Comparison

Comparing performance, the real model performed best among the tested models (Figure 7c). Since the real model was trained with images captured in the actual RAS environment and had sufficient data from which to learn, the model performed well while deployed in similar conditions. Unlike the real model, the virtual model was trained solely with virtual images, and although these images were simulated to mimic fish schooling, it was difficult to match the exact body texture, orientation, fish-to-fish color, and shape variation. These differences may have contributed to the inferior performance of the virtual model. Despite the poor performance of the virtual model, this model was able to detect the whole fish in the frame. Therefore, for an application where partial fish detection is not important and whole fish alone is of interest (e.g., fish biomass estimation), virtual models could be utilized. Despite a 4% difference in the mAP score and a difference of 0.04 in the F1 score between the real and M6 mixed model (Figure 7c,d), the latter performed satisfactorily in terms of partial and whole fish detection, as discussed in Section 3.2. A representative training dataset attained by supplementing real images in the training set of mixed models assisted in improving model robustness.

### 3.4. Time Cost Analysis

This analysis indicated that the time cost required to train the virtual model was considerably shorter than that for the real and mixed models (Table 2). The time required to annotate 700 virtual images was around 216 times shorter than that for the manual annotation of the same number of real images. The automated annotation of the virtual images aided in achieving a substantial reduction in the annotation time. Additionally, the virtual model’s training was terminated at lower epochs, as described in Section 3.1.1. Therefore, the training time for the virtual model was also reduced by half as compared to the real model. Overall, the total time cost required to train the virtual model was around 42 times shorter than that for the real model. While the virtual model’s training was much faster and more labor-efficient than that of the real model, the model performance of the former was not satisfactory (Figure 7c,d). Further investigation indicated that the annotation time for the M6 mixed model was around 10 times shorter than that for the real model. Since 90% of the training images (i.e., 630 virtual images) were annotated automatically, the annotation time for the M6 model reduced substantially. Moreover, the training time for M6 was also marginally shorter than that for the real model. The total time cost required to train the mixed model was seven times shorter than that for the real model, with the two models showing nearly similar model performance, as described in Section 3.2.

Notably, the data acquisition time for real images and the time spent in generating the simulated datasets were not included in the time cost analysis. It was assumed that data acquisition/simulation is a one-time process and that it will be reused for future projects. Additionally, virtual models can be easily replicated for various species cultured in aquaculture tank systems. Overall, the models trained with virtual images substantially reduced the training time, which means they can assist in the rapid deployment of fish detection models for aquaculture applications.

## 4. Conclusions

This study was conducted to assess the feasibility of using computer-simulation-generated virtual images to train a fish detection model for a real RAS environment. The following are the major conclusions derived from the presented investigation:

1. The virtual model trained solely with simulated images did not perform satisfactorily in partial fish detection; however, replacing small numbers of virtual images from the training dataset with real images significantly improved model performance. The M6 mixed model trained with 630 virtual and 70 real images achieved a satisfactory mAP of 91.8% and an F1 score of 0.87, and it precisely detected whole and partial fish in an actual RAS environment.
2. The automated annotation considerably reduced the annotation time for virtual images. This resulted in a seven-fold reduction in total training time cost for the M6 mixed model. Overall, virtual simulation can assist in developing a rapid and robust fish detection model for aquaculture applications.

While this study focused specifically on underwater fish detection, a similar approach can be adapted to train Machine Learning models with the help of simulated data for various purposes, such as detecting disease conditions (e.g., fin erosion, musculoskeletal deformities, cataracts, etc.), tracking fish maturity, monitoring fish schooling behavior, and other welfare indicators. Moreover, the present study aimed to train a fish detection model for a real RAS environment; however, a similar approach can be also adopted for other aquaculture production systems. Recent advancements in Large Language Model (LLM)-assisted image generation tools have provided the capability to efficiently generate extensive image datasets for Machine Learning model training without sophisticated simulation software. Therefore, future research should explore the potential of AI-generated datasets to train ML models to address challenges in the aquaculture industry.

## Figures and Tables

**Table 1:** Fish detection models trained with virtual-, real-, and mixed-image datasets.

Training Dataset	Model Name	Virtual to Real Image Proportion	Number of Training Images	
			Virtual	Real
Virtual images	Virtual	100:0	700	0
Virtual and real images	* M1	99:1	693	7
	M2	98:2	686	14
	M3	96:4	672	28
	M4	94:6	658	42
	M5	92:8	644	56
	M6	90:10	630	70
	M7	75:25	525	175
	M8	50:50	350	350
Real images	Real	0:100	0	500

* M represents mixed models trained with varying proportions of virtual and real images.

**Table 2:** The total time costs of virtual, mixed, and real model training.

Model Name	Annotation Time (s)		Training Time (s)	Total Training Time Cost (s) *
	Virtual Image	Real Image		
Virtual	330.0	0.0	1429.2	1759.2
Mixed (M6)	297.8	7140.0	2750.4	10,187.4
Real	0.0	71,400.0	2826	74,226

* reported time for annotating and training 700 images.

## Data Availability

Data will be made available on request.
